# Dynamic full-field optical coherence tomography for live-cell imaging and growth-phase monitoring in *Aspergillus fumigatus*

**DOI:** 10.3389/fcimb.2023.1183340

**Published:** 2023-07-12

**Authors:** Thomas Maldiney, Dea Garcia-Hermoso, Emilie Sitterlé, Jean-Marie Chassot, Olivier Thouvenin, Claude Boccara, Mathieu Blot, Lionel Piroth, Jean-Pierre Quenot, Pierre-Emmanuel Charles, Vishukumar Aimanianda, Bianca Podac, Léa Boulnois, Frédéric Dalle, Marc Sautour, Marie-Elisabeth Bougnoux, Fanny Lanternier

**Affiliations:** ^1^ Department of Intensive Care Medicine, William Morey General Hospital, Chalon-sur-Saône, France; ^2^ Lipness Team, Institut national de la santé et de la recherche médicale (INSERM) Research Centre Lipides, Nutrition, Cancer - Unité mixte de recherche (LNC-UMR)1231, University of Burgundy, Dijon, France; ^3^ Institut Pasteur, Université Paris Cité, National Reference Centre for Invasive Mycoses and Antifungals, Translational Mycology Research Group, Mycology Department, Paris, France; ^4^ Centre hospitalier universitaire (CHU) Necker Enfants Malades, Assistance publique - Hôpitaux de Paris (AP-HP), Institut Pasteur, Paris, France; ^5^ Institut Langevin, École supérieure de physique et de chimie industrielles de la ville de Paris (ESPCI) Paris, Paris Sciences & Lettres (PSL) University, Centre national de la recherche scientifique (CNRS), Paris, France; ^6^ Infectious Diseases Department, Dijon Bourgogne University Hospital, Dijon, France; ^7^ Institut national de la santé et de la recherche médicale (INSERM), CIC1432, Clinical Epidemiology Unit, Dijon, France; ^8^ Department of Intensive Care Medicine, Dijon Bourgogne University Hospital, Dijon, France; ^9^ Institut Pasteur, Université de Paris, Immunobiology of Aspergillus , Paris, France; ^10^ Medical Biology Laboratory, William Morey General Hospital, Chalon-sur-Saône, France; ^11^ Department of Parasitology/Mycology, Dijon Bourgogne University Hospital, Dijon, France; ^12^ Unité Mixte de Recherche Procédés Alimentaires et Microbiologiques (UMR PAM) A 02.102 Procédés Alimentaires et Microbiologiques, Univ. Bourgogne Franche-Comté, AgroSup Dijon, Dijon, France; ^13^ Institut national de recherche pour l'agriculture, l'alimentation et l'environnement (INRAE), Unité sous contrat (USC) 2019, Unité Biologie et Pathogénicité Fongiques, Paris, France; ^14^ Department of Infectious Diseases and Tropical Medicine, Necker-Enfants Malades Hospital, Assistance Publique-Hôpitaux de Paris, Paris, France

**Keywords:** dynamic full-field optical coherence tomography, *Aspergillus fumigatus*, invasive fungal infections, fungal metabolism, live-cell imaging

## Abstract

**Introduction:**

The diagnosis of cutaneous manifestations of deep mycoses relies on both histopathological and direct examinations. Yet, the current diagnostic criteria cannot prevent missed cases, including invasive aspergillosis, which requires the development of a novel diagnostic approach and imaging tools. We recently introduced the use of dynamic full-field optical coherence tomography (D-FF-OCT) in fungal diagnostics with a definition approaching that of conventional microscopy and the ability to return metabolic information regarding different fungal species. The present work focuses on subcellular dynamics and live-cell imaging of *Aspergillus fumigatus* with D-FF-OCT to follow the fungal growth stages.

**Methods:**

The *A. fumigatus* ATCC 204305 quality-control strain was used for all imaging experiments, following incubation times varying between 24 and 72 h at 30°C in a humidified chamber on Sabouraud dextrose agar. Fungal growth was subsequently monitored with D-FF-OCT for up to 5 h at room temperature and following the pharmacological stress of either voriconazole, amphotericin B, or caspofungin gradient concentration.

**Results:**

D-FF-OCT images allow not only the visualization of intracellular trafficking of vacuoles but also an evolving dynamic segmentation of conidiophores depending on the chronological development and aging of the hyphae or the effect of antifungal treatment. The same applies to conidial heads, with the most intense D-FF-OCT signal coming from vesicles, revealing a changing dynamic within a few hours only, as well as complete extinction following subsequent drying of the Sabouraud dextrose agar.

**Discussion:**

These results provide additional data on the ability of D-FF-OCT to monitor some of the main life cycle processes, dynamics, and intracellular trafficking of vacuoles in *A. fumigatus*, with or without the effect of pharmacological stress. Such complementary metabolic information could help both clinicians and microbiologists in either mechanistic studies toward experimental mycology or the development of a potential D-FF-OCT-guided diagnosis of superficial fungal infections.

## 1 Introduction

Superficial fungal infections refer to a rather large and heterogeneous class of fungal diseases in both immunocompromised and non-immunocompromised patients. These range from dermatophyte infections (Schwartz, 2004) to more complex cutaneous manifestations of invasive fungal infections (IFIs) (Shields et al., 2019) and even fungal keratitis (Olivier et al., 2022). In the case of invasive aspergillosis, the existence of specific diagnostic criteria (Donnelly et al., 2020) may be useful to identify a given population in view of future clinical trials. However, these criteria are also associated with real-life diagnostic problems and numerous missed cases when compared with postmortem reports (Danion et al., 2019). Thus, the optimization of IFI and superficial fungal infection diagnosis should not only associate a better communication between clinicians and biologists but also count on the development of and wider access to novel routine technologies with the ability to exploit additional information, such as high-resolution direct examination or even metabolic identification of fungi (Khan et al., 2022).

Until now, the visualization of subcellular structures and dynamics within filamentous fungi has mainly referred to a standard scientific method to better understand the physiological and developmental processes *in vivo* (Hickey et al., 2004). Such metabolic monitoring of living cells in medical mycology usually relies on combining confocal microscopy systems with high spatial resolution (St. Croix et al., 2005) using specific fluorescent dyes or conjugates that can highlight different cellular compartments (Lichius and Zeilinger, 2019). Advanced computer skills are then required to process all recorded data and produce a high-resolution multicolored image (Gupta et al., 2022). When applied to the visualization of *Aspergillus fumigatus* subcellular compartments (cell wall, membrane, mitochondria, or vacuoles), the potential of this kind of imaging protocol to provide valuable metabolic information regarding several *in vitro* applications has been demonstrated, notably in view of the future development and testing of antifungal molecules (Pfister et al., 2020).

However, the relative complexity of these multistaining experiments still largely hampers any direct transposition of such methods to clinical microbiological routine practice for applied diagnostics in medical mycology. The shortfall is notable compared with the growing impact of rapid testing technologies such as matrix-assisted laser desorption/ionization time of flight (MALDI-TOF) (Medina et al., 2022) and molecular-based diagnosis (Wickes and Wiederhold, 2018). The European Organization for Research and Treatment of Cancer and the Mycoses Study Group Education and Research Consortium (EORTC/MSGERC) produced a detailed revision and update regarding the consensus definitions and diagnostic criteria for IFI. This confirms the importance of both microscopic examination and histopathology as gold standards for the classification of the proven IFIs (Donnelly et al., 2020). Recently, our group introduced the first results demonstrating the use of dynamic full-field optical coherence tomography (D-FF-OCT) as a complementary tool in fungal diagnostics (Maldiney et al., 2022).

D-FF-OCT relies on a specific kind of interference microscopy with a broadband light source. This provides both high-resolution and subcellular metabolic contrast imaging of biological tissues without the need for any tissue sample preparation or coloration step (Apelian et al., 2016; Maldiney et al., 2020; Quénéhervé et al., 2021; Wang et al., 2022). When applied to the observation of different filamentous fungal species, D-FF-OCT makes it possible to identify multiple intracellular compartments with a definition approaching that of conventional microscopy. It can also retrieve novel metabolic information in relation to fungal subcellular dynamics (Maldiney et al., 2022). Contrary to our previous study, which only described D-FF-OCT preliminary images for three different fungal species, the present work intends to demonstrate how D-FF-OCT may serve live-cell imaging and monitoring of fungal growth stages by focusing on *A. fumigatus*.

## 2 Materials and methods

### 2.1 Aspergillus fumigatus strain

The *A. fumigatus* ATCC 204305 quality-control strain was used for all imaging experiments with the D-FF-OCT system. Solid cultures were grown following incubation times varying between 24 and 72 h at 30°C in a humidified chamber on Sabouraud dextrose agar (BioMérieux SA, Marcy-l’Etoile, France) before subsequent D-FF-OCT acquisition.

### 2.2 Antifungal susceptibility testing

Etest strips containing caspofungin, voriconazole, or amphotericin B and Roswell Park Memorial Institute RPMI agar (RPMI 1640, MOPS, L-glutamine, 2% glucose) were supplied by BioMérieux SA (Marcy-l’Etoile, France). Briefly, the minimum inhibitory concentration (MICs) of caspofungin, voriconazole, and amphotericin B were determined by using the EUCAST standardized methodology for the *A. fumigatus* ATCC 204305 quality-control strain by using the gradient concentration strip (GCS) method on RPMI agar.

### 2.3 D-FF-OCT standard and dynamic contrast imaging

As previously described (Ghouali et al., 2015; Maldiney et al., 2020; Maldiney et al., 2022), D-FF-OCT images were acquired at room temperature with the Light-CT Scanner apparatus accessible at Langevin Institute (LLTech – Aquyre Biosciences). As described in **Supplementary Figure S1** and **Supplementary Table**, the system associates a conventional halogen light source with short temporal coherence length and a set of 10× water immersion microscope objectives with a numerical aperture of 0.3 in a so-called Linnik configuration to reach a final axial and transverse resolution of approximately 1–1.5 µm.

For each acquisition, rectangular patches of approximately 2 cm long and 1 cm wide either Sabouraud dextrose agar or RPMI agar-grown *A. fumigatus* were placed on the sample holder of the FF-OCT apparatus. Fungal growth was subsequently monitored following both standard full-field illumination (FF-OCT) and subsequent dynamic contrast imaging (D-FF-OCT) for up to 5 h at room temperature without the addition of any dye or coloration step. Note that the illumination was stopped between different acquisitions for time series. Images were acquired with a 1-µm axial pace, up to a depth of 50 µm, and reconstructed with ImageJ software.

### 2.4 Image analysis

ImageJ 1.50e software was used to analyze and reconstruct all D-FF-OCT-acquired image stacks. Axial z-stacking of multiple images was reconstructed with the “Z-project” function following a “standard deviation” projection type for D-FF-OCT acquisitions and a “maximum intensity” projection type for standard FF-OCT images. The thickness of the axial z-stacking varied between 5 and 50 µm, depending on the information required. Three-dimensional reconstructions of either FF-OCT or D-FF-OCT acquisitions were accessible with the “Volume Viewer” plugin. The background signal was voluntarily inverted from black to white for the visualization of the three-dimensional D-FF-OCT projection to facilitate the interpretation of contrast within conidial heads. A contrast-based ImageJ protocol called the “Plot Profile” function allowed length measurements of each fungal compartment for both standard and dynamic contrast images. Finally, as stated in our previous study regarding the specific analysis of D-FF-OCT images (Maldiney et al., 2022), a fast Fourier transform program was used to study the time series corresponding to each pixel behavior. The power spectrum density was split into three parts of equal energy, each matching one of the three main colors of the composite RGB image. As such, the high frequencies (fast movements) correspond to the red color, the medium frequencies (intermediate movements) correspond to the green color, and the low frequencies (slow movements) correspond to the blue color.

## 3 Results

### 3.1 In-depth D-FF-OCT imaging

**Figure 1:**
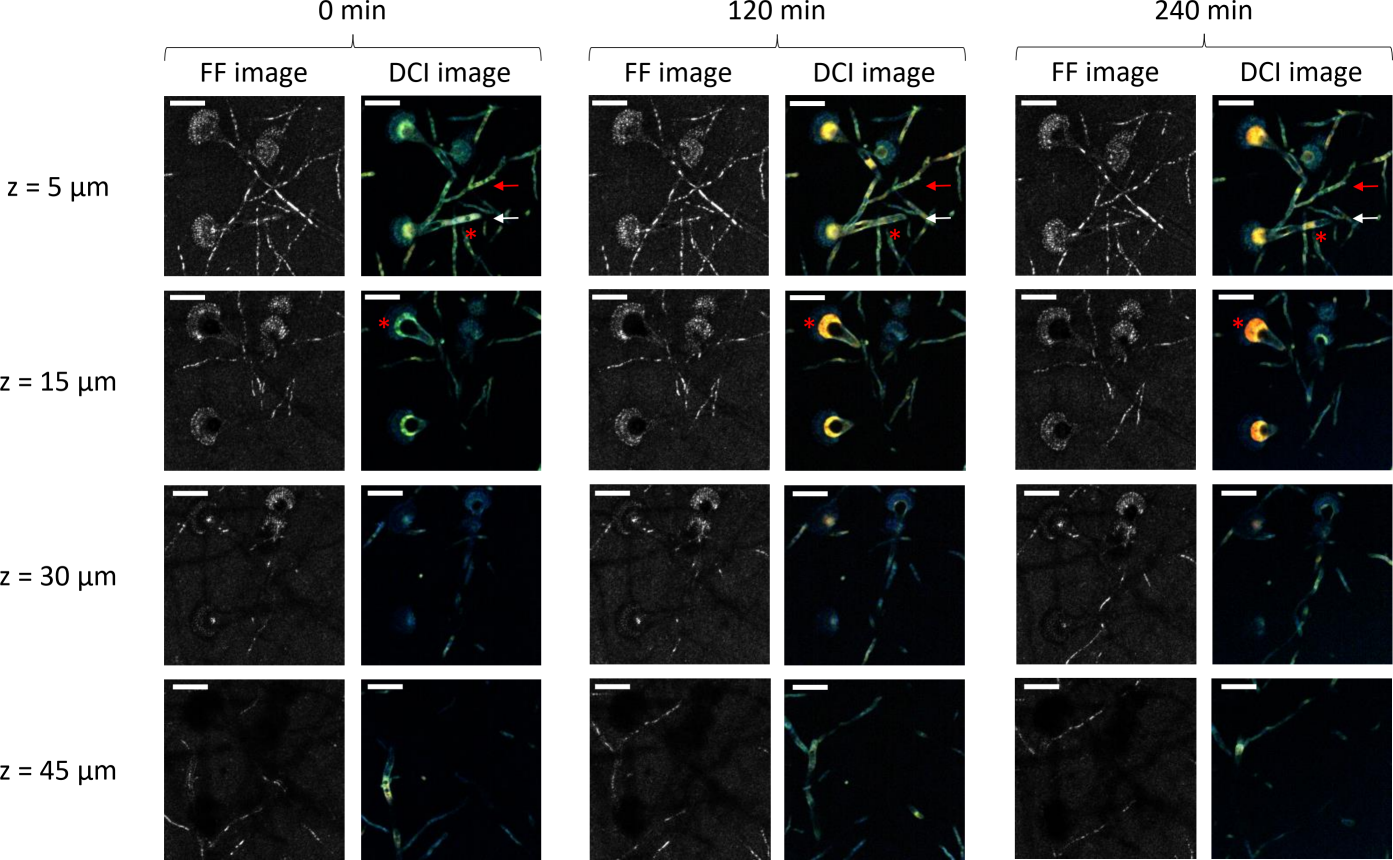
In-depth full-field (FF)- and dynamic full-field optical coherence tomography (D-FF-OCT) imaging of *Aspergillus fumigatus* growth on Sabouraud dextrose agar for 240 min following a 24 h incubation at 30°C in a humidified chamber. Each image corresponds to a 5-µm-deep axial Z-projection of the same region of interest at different times (0, 120, and 240 min) and depths (5, 15, 30, and 45 µm). The scale bar represents 25 µm. The composite RGB DCI image translates each pixel movement as a color (red for high frequencies/fast movements, green for medium frequencies/intermediate movements, and blue for low frequencies/slow movements). The red arrows show a conidiophore with a stable DCI signal in time. The white arrows show a conidiophore with an evolving DCI signal in time. The red asterisks identify intracellular vacuoles.

**Figure 1** shows the in-depth D-FF-OCT images of *A. fumigatus* within Sabouraud dextrose agar at different times and depths. Regardless of the depth, a standard FF-OCT image of conidiophores shows what appears to be thin and septate hyphae with a diameter ranging from 2 to 4 µm. Apart from these most intense filamentous segments, phialides and conidia also give a relatively bright FF-OCT signal compared with other compartments of the conidial head, notably the vesicle. When going deeper within the Sabouraud dextrose agar, in-depth FF-OCT imaging allows the visualization of different levels of *A. fumigatus* uniseriate and columnar conidial heads of approximately 30–50 µm. Comparison of the most superficial (5 µm) and the deepest (45 µm) FF-OCT projections indicates the ability of in-depth imaging to follow the orientation of the conidiophore, but there was also a slight loss in global FF-OCT signal intensity. Although time does not seem to have any influence on the region of interest presented in **Figure 1**, with a global stability of the FF-OCT signal within all compartments of both the conidiophore and conidial head at 0, 120, and 240 min, the D-FF-OCT images highlight a different trend.

Firstly, dynamic contrast imaging within the two most superficial depths (5 and 15 µm) of the Sabouraud dextrose agar culture gives complementary information regarding conidiophores and conidial heads. Here, the signal from the hyphae is slightly larger than the one obtained with FF-OCT (diameter ranging between 4 and 6 µm), and the metabolic segmentation intensity is significantly different from the one identified with FF-OCT (red and white arrows). In numerous segments of conidiophores, the D-FF-OCT signal appears brighter, while the FF-OCT signal is almost completely extinct. In addition, further monitoring of the D-FF-OCT signal after 120 and 240 min shows an evolution of both intensity and color coming from the identified conidiophores (white arrow), compared with others (red arrow). It also allows the visualization of intracellular vacuole modifications (morphological aspect and localization) within both conidiophores and conidial heads (red asterisks). The comparison of DCI signals coming from conidial heads located in the two most superficial layers (5 and 15 µm) of the Sabouraud dextrose agar culture at 0, 120, and 240 min confirms two specific aspects of dynamic contrast imaging. Firstly, the D-FF-OCT signal is more intense within vesicles from the conidial heads than in any other fungal compartments. In contrast, phialides and conidia show a high FF-OCT signal but only a low dynamic signal. Secondly, the color of the D-FF-OCT signal from the vesicles clearly changes from green to red after 240 min, reflecting faster movements inside the vesicle over time.

### 3.2 Three-dimensional and whole-volume D-FF-OCT imaging

**Figure 2:**
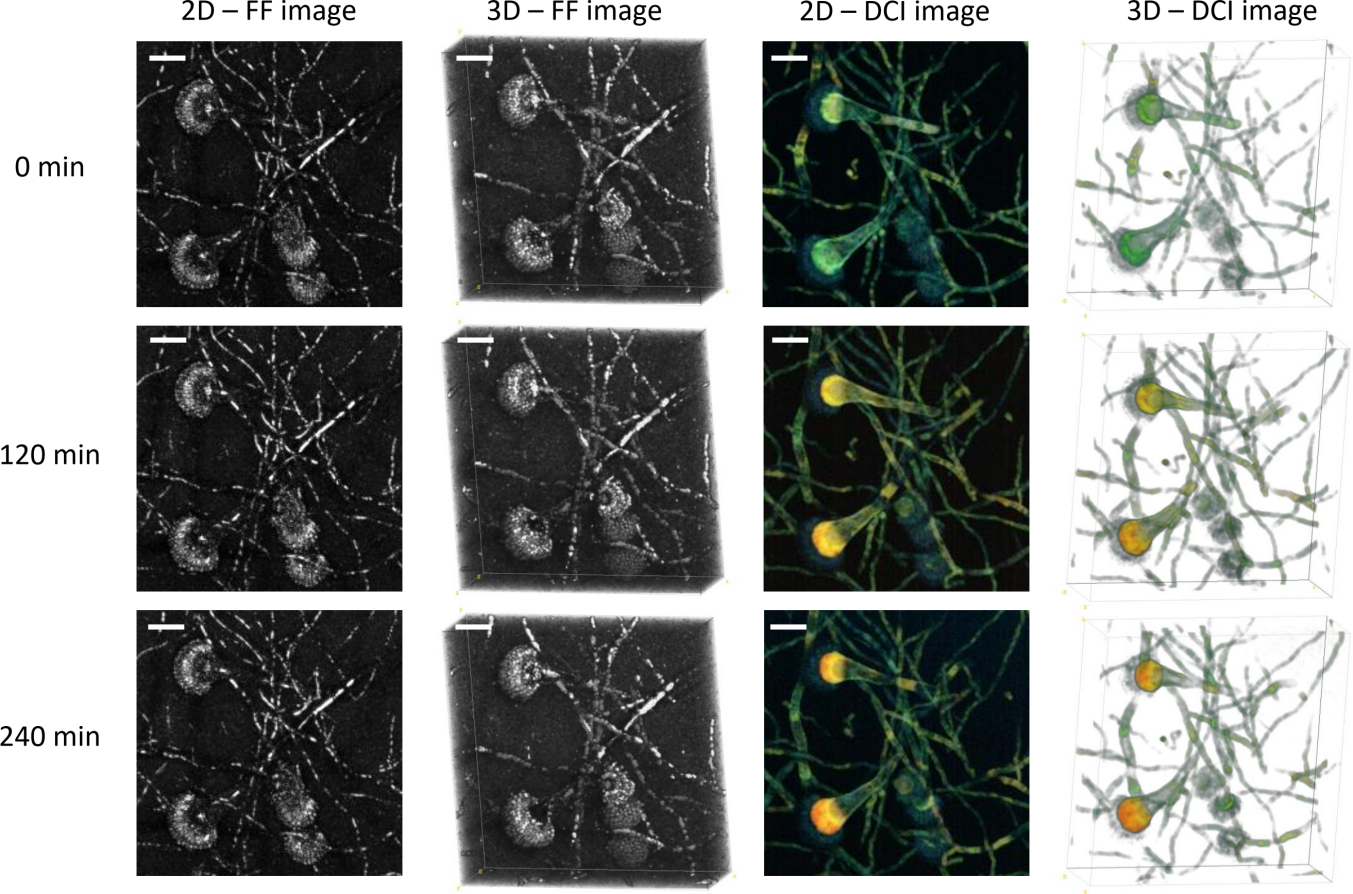
Three-dimensional FF- and D-OCT imaging of *Aspergillus fumigatus* growth on Sabouraud dextrose agar for 240 min following a 24 h incubation at 30°C in a humidified chamber. Each 2D image corresponds to a 50-µm-deep axial Z-projection of the same region of interest at different times (0, 120, and 240 min). Each 3D image corresponds to a 50-µm-deep axial volume reconstruction of the same region of interest at different times (0, 120, and 240 min). The scale bar represents 25 µm. The composite RGB DCI image translates each pixel movement as a color (red for high frequencies/fast movements, green for medium frequencies/intermediate movements, and blue for low frequencies/slow movements). The background noise was modified to white for the three-dimensional D-OCT reconstruction to enhance the visualization of dynamic contrasts within the conidial heads.

**Figure 2** shows the results of the three-dimensional D-FF-OCT and whole-volume reconstructions of the same region of interest as the one displayed in **Figure 1**. The two-dimensional whole-volume 50-µm-deep axial Z-projection confirms the morphological aspects accessible with in-depth standard and dynamic contrast imaging. As described previously with the information from all depths gathered on the same axial Z-projection, most of the FF-OCT signal comes from thin and septate hyphae as well as phialides and conidia from the conidial heads. The three-dimensional reconstruction allows the visualization of *A. fumigatus* uniseriate, spherical, and columnar conidial heads as part of a whole-volume image of approximately 50 µm × 180 µm × 180 µm. In addition, the two-dimensional and three-dimensional dynamic contrast reconstructions provide a global confirmation regarding the added value of a D-FF-OCT signal and its ability to provide additional information in comparison with FF-OCT. Notably, a D-FF-OCT signal reveals slightly larger conidiophores with a specific dynamic segmentation and color variation ranging from blue to red, depending on the fungal compartment and moment in time. **Supplementary Figure S2** identifies a similar dynamic segmentation within the conidiophore with a different intensity (from pale to bright), color (from blue to yellow), and segmentation (progressive segmentation from the first to the fifth region of interest) depending on the conidiophore and location inside the Sabouraud dextrose agar. Indeed, the modification of background noise from black to white for the three-dimensional D-OCT reconstructions from **Figure 2** enhances the visualization of dynamic contrasts within the conidial heads and confirms a pale blue signal coming from the phialides. This is opposed to the vesicles that clearly display the most intense dynamic signal, turning from blue–green (0 min) to yellow (120 min) and finally red (240 min). These late results are largely confirmed by live monitoring of the same vesicle up to 360 min after the beginning of the acquisition (**Supplementary Video 1**).

### 3.3 Whole-volume D-FF-OCT image analysis

**Figure 3:**
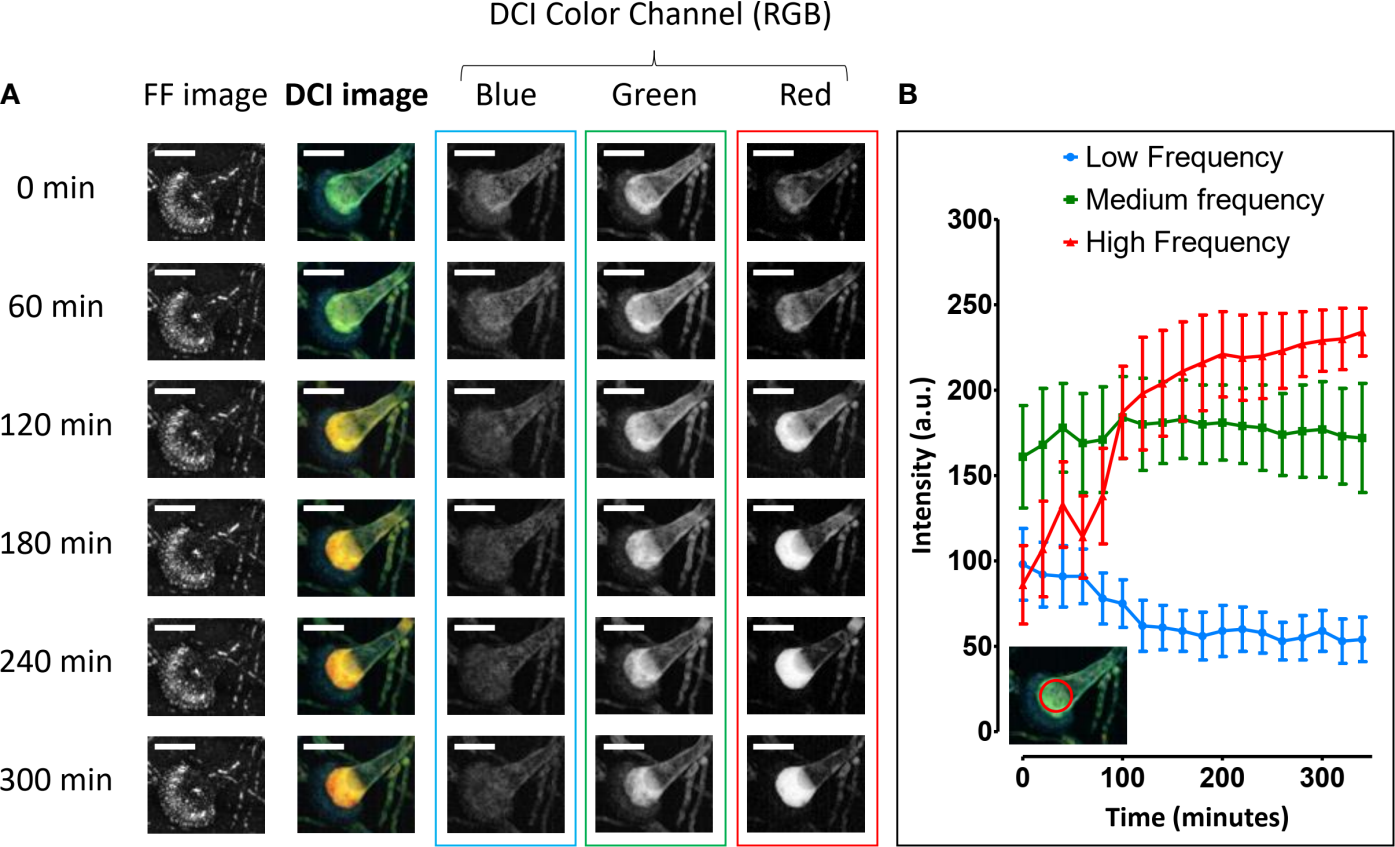
Whole-volume D-OCT image analysis of *Aspergillus fumigatus* growth on Sabouraud dextrose agar for 300 min following a 24 h incubation at 30°C in a humidified chamber. The three RGB DCI image color channels are displayed as 50-µm-deep axial Z-projections of a conidial head at different times (0, 60, 120, 180, 240, and 300 min): red for high frequencies/fast movements, green for medium frequencies/intermediate movements, and blue for low frequencies/slow movements **(A)**. The corresponding histogram displays the evolution of each color channel intensity signal within the red circular region of interest located inside the conidial head vesicle for approximately 5 h **(B)**. The scale bar represents 25 µm.

**Figure 3** further analyzes the evolution of the intensity and nature of the D-FF-OCT signal coming from the vesicle of a conidial head. It compares both the FF-OCT and D-FF-OCT signals to the three split RGB DCI image color channels. Each image corresponds to a 50-µm-deep axial Z-projection. **Figure 3A** displays the FF-OCT image, the D-FF-OCT image, and all three RGB D-FF-OCT image color channels (blue, green, red) at different times (0, 60, 120, 180, 240, and 300 min). Firstly and as noted from the data displayed in **Figures 1**, **2**, **3A** does not show any evolution of the FF-OCT signal from 0 to 300 min. On the contrary, dynamic contrast imaging of the conidial head confirms a clear evolution of the D-OCT signal, which turns from green to red after 300 min (faster movements). Such a trend in the evolution of the dynamic contrast within the vesicle is further analyzed with the decomposition of all RGB DCI image color channels. This reveals both a progressive decline of the blue channel contribution and a significant increase in the signal intensity coming from the red channel. The data from **Figure 3A** also confirm the relative stability of the signal from the green channel with time.

**Figure 3B** presents a semiquantitative analysis of the signal coming from each RGB DCI image color channel after using a fast Fourier transform program to study the time series corresponding to each pixel behavior (live monitoring for approximately 360 min). The decision to divide the whole spectrum by identifying red for high frequencies (fast movements), green for medium frequencies (intermediate movements), and blue for low frequencies (slow movements) results in the histogram displayed in **Figure 3B**. This shows that despite a global stability of the contribution due to medium frequencies, the evolution of the dynamic contrast within the vesicle is mainly the result of two concomitant tendencies. Firstly, there is a growing pre-eminence of high frequencies that translates into a higher intensity of the signal coming from the red channel, when compared with either the green (medium frequencies) or blue (low frequencies) channels. Secondly, there is a significant decrease in the contribution of slow movements to the final dynamic contrast signal.

### 3.4 D-FF-OCT and live monitoring of developmental processes

**Figure 4:**
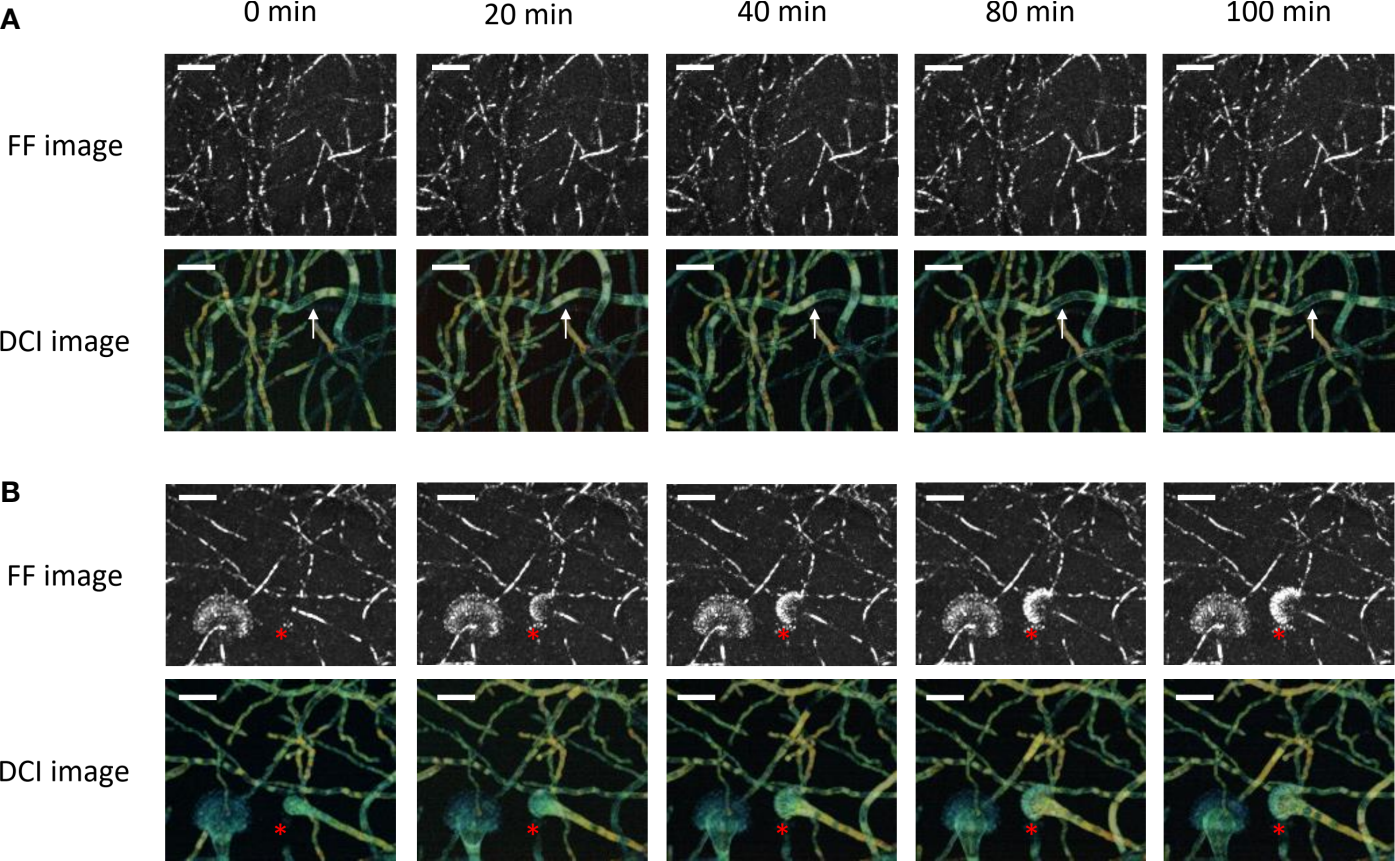
Whole-volume FF- and D-OCT live imaging of *Aspergillus fumigatus* growth on Sabouraud dextrose agar for 100 min following a 24 h incubation at 30°C in a humidified chamber. The images display a 50-µm-deep axial Z-projection of the conidiophores **(A)** and conidial heads **(B)** at different times (0, 20, 40, 80, and 100 min). The scale bar represents 25 µm. The composite RGB DCI image translates each pixel movement as a color (red for high frequencies/fast movements, green for medium frequencies/intermediate movements, and blue for low frequencies/slow movements). The white arrows show a conidiophore with vacuole trafficking and evolving DCI signal in time. The red asterisks locate the formation of the conidial head.

**Figure 4** and **Supplementary Figure S3** give the results from the whole-volume D-FF-OCT live monitoring of the developmental processes of a conidiophore and conidial head. The data from **Figure 4A** focus on the development of a conidiophore within Sabouraud dextrose agar for 100 min. As described in **Figures 1**–**3**, the live monitoring of the conidiophore FF-OCT signal from this region of interest does not seem to vary with time. On the contrary, D-FF-OCT images from **Figure 4A** and **Supplementary Video 2** show the serpentining evolution of the dynamic contrast signal, going from one end of the conidiophore, marked with a white arrow, to the other. This change in the D-FF-OCT signal is not common to all conidiophores from the region of interest. However, it also demonstrates the ability of dynamic contrast to allow live monitoring of intracellular vacuole trafficking, coalescence, and morphological modifications within the conidiophores. Interestingly, **Supplementary Video 1** shows that the vacuoles move away from the conidial head. **Supplementary Figure S3** highlights a different region where the hyphae appear thinner, with D-OCT images displaying a 50-µm-deep axial Z-projection of growing conidiophores within the same volume of Sabouraud dextrose agar at different times (0, 20, 40, 60, 80, and 100 min). The white arrows in **Supplementary Figure S3** point to a conidiophore growing at a speed of approximately 50 µm/h, with a green DCI signal that is rather homogeneous when compared with the metabolic segmentation displayed in **Supplementary Figure S2**.

The D-FF-OCT images from **Figure 4B** show the progressive formation of a conidial head over 100 min. As previously reported in **Figures 1**, **2**, **4A**, the development of phialides is most clearly visualized with FF-OCT, without significant changes in the signal coming from the hyphae or vesicle (red asterisks). Subsequent monitoring of the D-FF-OCT signal gives live access to the growth of the vesicle and phialides. It also returns the dynamic contrast signal modifications within each fungal compartment, starting from the conidiophore, whose D-FF-OCT signal changes from yellow to orange after 100 min, reflecting faster movements. This is accompanied by the migration of several vacuoles toward the distal end of the growing conidial head inside the vesicle (red asterisks and **Supplementary Video 3**). Indeed, the evolution of the vesicle dynamic contrast, from green to yellow, also reflects a significant increase in movement frequencies throughout the development process. As such, the newly formed phialides displaying a green D-FF-OCT signal can be compared with the pale blue—i.e., slower —signal from phialides in **Figure 2**.

**Supplementary Figure S4** presents the results from the whole-volume FF- and D-FF-OCT imaging of *A. fumigatus* conidiophores within Sabouraud dextrose agar following different incubation periods (24, 48, and 72 h). The FF-OCT images show that longer incubation periods at 30°C are associated with a progressive modification of the hyphal forms. These become both thicker and bendier when compared with the compartments described in **Figures 1**, **2**, **4**. In addition, this apparent change in conidiophore structure goes alongside a loss of dynamic contrast segmentation, with the hyphae evolving from a green to a yellow segmented aspect after a 24-h incubation period, to an almost unsegmented and homogeneous blue following a 48-h incubation period. Note that a longer incubation period of up to 72 h at 30°C induces a complete loss of the D-FF-OCT signal.

Finally, **Supplementary Figures S5–S7** show the whole-volume FF- and D-OCT images of *A. fumigatus* conidiophores within RPMI agar depending on the distance to an Etest voriconazole, amphotericin B, or caspofungin strip. First, the MICs of voriconazole, amphotericin B, and caspofungin were determined to be 0.094, 0.38, and 0.016 mg/L, respectively. These results altogether confirm that the *A. fumigatus* ATCC 204305 strain can be considered susceptible to all three antifungal treatments. Then, the comparison of **Supplementary Figures S5–S7** shows that all antifungal treatments significantly inhibit the growth of *A. fumigatus*. **Supplementary Figures S5, S6** display a similar effect of voriconazole and amphotericin B, both responsible for a distinct decrease of hyphal concentration, as seen from either the FF- or D-OCT image, associated with a shift of the dynamic contrast signal, which turns from warm green–yellow to pale blue–green (slower movements) upon an increase of fungicidal concentration. Data from **Supplementary Figure S7** suggest the same behavior with caspofungin regarding the evolution of the D-OCT signal. However, the D-FF-OCT signal enlightens a more compact aspect of *A. fumigatus* microcolonies in the regions of interest with higher caspofungin concentrations. When compared with the pattern displayed in the region of interest with low caspofungin concentration, these microcolonies appear with dense and packed hyphae showing predominant blue D-OCT signal (slow movements).

## 4 Discussion

For the first time, the present study demonstrates the use of D-FF-OCT imaging for live-cell imaging and monitoring of the developmental processes in *A. fumigatus*. As previously reported from preliminary static acquisitions of several filamentous species (Maldiney et al., 2022), D-FF-OCT returns two pieces of complementary tomographic information. These concern both the structure and dynamic contrast of the main fungal compartments composing *A. fumigatus.* They may serve both future microbiological applications and potential diagnostic purposes in routine clinical practice related to superficial fungal infections.

### 4.1 In-depth D-FF-OCT imaging

Confocal laser scanning microscopy (CLSM) has been conventionally used for years to monitor developmental processes within filamentous fungi (Czymmek et al., 1994; Mouriño-Pérez and Roberson, 2015; Du et al., 2016). However, in comparison, in-depth D-FF-OCT imaging gives access to both structural and dynamic multidimensional microscopy with the ability to monitor the development and growth of *A. fumigatus* in the whole volume of a solid Sabouraud dextrose agar. Indeed, the data from **Figure 1** demonstrate the ability of such high-resolution interference microscopy to provide full-field images as optical sections along the axial *Z*-axis without the need for concomitant physical sectioning or preparation of the sample. In addition to the observations from recent preliminary experiments (Maldiney et al., 2022), the current spatial resolution of the system allows an in-depth visualization of the structures of the main *A. fumigatus* compartments (conidiophores, septa, vacuoles, vesicles, phialides, and conidia). It also permits their respective dynamic contrast with an increased temporal resolution, thus opening access to subsequent acquisitions at different times. Such a report of in-depth D-FF-OCT-based monitoring of *A. fumigatus* dynamics is, to our knowledge, the first attempt to use low coherence interference microscopy for live-cell imaging in medical mycology (Hickey and Read, 2009; Islam et al., 2017; Pfister et al., 2020; Wang et al., 2022).

### 4.2 Whole-volume D-FF-OCT imaging

The results from ImageJ processing of D-FF-OCT optical sections demonstrate the potential of both three-dimensional and whole-volume reconstructions to gather the information located within all discrete Z-levels in a single image returning either FF-OCT or D-OCT signals. A side-by-side comparison of D-FF-OCT and CLSM technologies is displayed in the **Supplementary Table**. Apart from conventional CLSM (Schlafer et al., 2018; Maslov et al., 2021) and the new light sheet fluorescence microscopy (LSFM) (Amich et al., 2020; Henneberg et al., 2021), D-FF-OCT is revealed to be one of the few imaging modalities based on optical imaging to rapidly provide such expertise in both three-dimensional and whole-volume descriptions of *A. fumigatus* developmental processes, with the ability to associate concomitant structural and metabolic information. In CLSM and LSFM, the metabolic subcellular contrast mainly comes from live fluorescence imaging following the use of specific fluorescent dyes and conjugates, as well as the well-known autofluorescence process (Lichius and Zeilinger, 2019; Amich et al., 2020; Pfister et al., 2020). In contrast, the dynamic signal from D-FF-OCT is to date widely believed to be the reflection of the active transport of intracellular organelles (Apelian et al., 2016; Scholler et al., 2020). When considering future microbiological or routine clinical applications, the main consequences and advantages of live dynamic contrast imaging with D-FF-OCT are that it requires neither preparation, coloration, or a fixation step of the sample before acquisition nor any recourse to a specific staining with fluorescent siderophores.

The visualization of bright FF-OCT signals indicates the presence of more scattering structures, either because their shape is different or their size or refractive index is bigger. As the refractive index typically scales linearly with the local dry mass (Welzel, 2001; Zangle and Teitell, 2014), a brighter FF-OCT signal might very likely be associated with either heavier or more abundant conidial cell wall proteins, as well as branched polysaccharides and 1,8-dihydroxynaphthalene (DHN) melanin (Baltussen et al., 2020) as reported from the molecular mechanisms of conidial germination in *Aspergillus* spp. described by Baltussen et al. (2020). Indeed, the postprocessing steps describing the optical route from the D-FF-OCT acquisitions to the dynamic contrast image have already been reported elsewhere as a combination of mean frequency, frequency bandwidth, and standard deviation of the amplitude signal (Apelian et al., 2016; Scholler et al., 2020). Thus, to facilitate the interpretation of the D-OCT signal, **Figure 3** focuses on the example of a conidial head and its dynamic contrast within the vesicle to translate the mean frequency of each pixel as a combination of colors from the DCI color channels (RGB).

The significant increase and the pre-eminence of fast movements recorded from the D-OCT signal are associated with a relative decline of slow movements inside the same vesicle. These results are to be understood alongside those from **Figure 4**, which describes the progressive live formation of a conidial head characterized by faster movements within the vesicle and migration of multiple vacuoles toward the hyphal tip. A comparison of such dynamic contrasts within conidiophores from **Figure 4** and **Supplementary Figures S2, S3** to the specific CLSM-based co-staining described in the work of Pfister et al. opens access to possible speculations regarding the interpretation of the present DCI signal in relation to the fungal metabolism. Interestingly, the present metabolic segmentation of conidiophores with D-FF-OCT could either match the one from [Fe]DAFC-BODIPY, -Cy5, and -SiR, which preferentially stained longitudinal structures reminiscent of fungal mitochondria, or from [Fe]DAFC-FITC that accumulated in the cytoplasm (Pfister et al., 2020). In both cases, such observations would confirm the general hypothesis in favor of a D-FF-OCT signal reflecting mitochondrial activity or cellular organelle movements (Scholler et al., 2020). Following a similar reasoning, the morphological aspect of intracellular vacuoles accessible in negative contrast from D-FF-OCT images largely corresponds to the one revealed by [Fe]DAFC-Ocean Blue and -NBD (Pfister et al., 2020).

### 4.3 D-FF-OCT and live monitoring of developmental processes

When looking at the results in **Supplementary Figure S4**, it may be of particular interest to transpose these metabolic conjectures regarding the nature of the D-OCT signal to the development and growth of *A. fumigatus* following incubation periods longer than 24 h. Indeed, the progressive structural and metabolic modifications of the hyphal aspects after an increase of the incubation period (up to 72 h) at 30°C finally result in both a significant change of the FF-OCT signal from conidiophores, which turns thick and bendy, and a complete extinction of the DFF-OCT signal. These morphological changes within fungal compartments were associated with visible drying of the Sabouraud dextrose agar patches, very certainly responsible for the concomitant suffering and death of the fungal cells. These results require further validation with a subsequent study of the potential impact of antifungal drugs on the D-FF-OCT signal. Nevertheless, they open a crucial alternative toward a future use of this technology to assess the viability of fungal cells without any preparation or coloration step.

To this end, the preliminary results from **Supplementary Figures S5–S7** appear to comfort the potential ability of D-FF-OCT dynamic contrast to reveal the metabolic impact of antifungal stress toward *A. fumigatus* growth. Indeed, the significant shift of the hyphal dynamic contrast signal under the pharmacological activity of the fungicidal molecules voriconazole and amphotericin B, respectively reported in **Supplementary Figures S5, S6**, associated with the distinct decrease of hyphal concentration confirmed with standard FF-OCT observations, acknowledges the results from the brightfield experiments by Lewis et al. studying *in vitro* dynamics of voriconazole and amphotericin B against *A. fumigatus* (Lewis et al., 2005) and those from Gangwar et al. showing the disintegration of mitochondria and nuclei following exposition to voriconazole and amphotericin B (Gangwar et al., 2006). The results with caspofungin associate not only the formation of packed and dense hyphae but also a significant slackening of their D-OCT dynamic contrast, whose intensity and frequency are both decreasing, altogether reflecting the well-known caspofungin-mediated paradoxical growth effect (Wagener and Loiko, 2017) occurring in *A. fumigatus* only for microcolonies in the regions of interest with higher caspofungin concentrations (**Supplementary Figure S7**), as in the work of Moreno-Velásquez et al. (2017).

Despite the wide range of possibilities considered from these results with D-FF-OCT live-cell imaging, the present study suffers from certain limitations that require additional developments and improvements. Firstly, the current spatial resolution of approximately 1–1.5 µm accessible with the commercial D-FF-OCT apparatus hampers any precise observation of fungal compartments and structures smaller than conidia. A possible alternative would be to create a dedicated experimental D-FF-OCT setup with high-magnification water immersion objectives, on the basis of the one described by Scholler et al. (2020). Secondly, the exact nature of both the FF- and D-OCT signals remains largely unclear in view of the rather complex developmental processes. As such, the present image analysis integrates neither the primary metabolism nor the fungal respiration of *A. fumigatus* (Willger et al., 2009). In addition, each specific growth mechanism comes with a molecular signature that identifies different metabolic phases, from the germination of conidia and mycelial growth to the development of conidial heads (van de Veerdonk et al., 2017; Baltussen et al., 2020). Investigating these signatures would possibly help to better understand the dynamic contrast images. The identification of a proper correspondence between a D-FF-OCT signal and a specific metabolic route will thus require a thorough comparison with organelle-specific staining and subsequent observation with CLSM. Finally, additional experiments will be required to compare the impact of multiple stress conditions, notably the action of different antifungal therapies (fungistatic and fungicidal drugs), on fungal cell viability and the properties of the D-FF-OCT signal (Shailaja et al., 2022).

Altogether, these unprecedented results prove how D-FF-OCT may serve not only live-cell imaging but also the monitoring of growth phases and rapid measurements of metabolic activity within *A. fumigatus*. Furthermore, image analysis would very certainly benefit from the use of mathematical models to better understand and characterize the pathogenesis of such *A. fumigatus* branching complexity monitored with D-FF-OCT (Rajković et al., 2019). Apart from the potential impact of such technology for mechanistic studies and experimental mycology, one should not minimize the possible advantages regarding clinical applications. Given the large amount of missed invasive aspergillosis cases retrieved from postmortem reports (Danion et al., 2019) and the known issues associated with its identification and management (Cole et al., 2017), both clinicians and microbiologists will need to implement novel imaging technologies as diagnostic tools in their routine healthcare practice and approaches to fungal characterization. The known abilities of D-FF-OCT to provide direct *in vivo* histopathological features (Dalimier and Salomon, 2012; van Manen et al., 2018), combined with the present results describing both structural and dynamic contrasts accessible for *A. fumigatus*, may serve new perspectives and studies. These could focus on the potential of D-FF-OCT for extemporaneous diagnosis of invasive aspergillosis following biopsy or surgical resection, as well as dermatophyte-associated or even direct invasive superficial fungal infections *in vivo*. Note that a recent report on real-time cellular imaging of the human cornea with dedicated and optimized non-contact OCT systems (Mazlin et al., 2020) might be of particular interest for the development and future improvements regarding *in vivo* diagnosis of fungal keratitis (Olivier et al., 2022). In addition to the conventional morphological aspect, the ability to record the live metabolic activity of any microbiological sample in routine practice, notably fungi, opens access to a novel kind of D-FF-OCT-based direct examination which is of particular interest in either mechanistic studies toward experimental mycology or for the development of a potential D-FF-OCT-guided diagnosis of superficial fungal infections.

## Data availability statement

The original contributions presented in the study are included in the article/**Supplementary Material**. Further inquiries can be directed to the corresponding author.

## Author contributions

TM, DG-H, M-EB, and FL designed the study, conducted the experiments, and performed the experimental works. TM wrote the manuscript. ES, J-MC, OT, CB, and LB performed the experimental works. TM, DG-H, ES, J-MC, OT, CB, MB, LP, J-PQ, PC, VA, BP, LB, FD, MS, M-EB, and FL contributed to the revision of the manuscript. TM, DG-H, M-EB, and FL supervised the project. All authors contributed to the article and approved the submitted version.
