# Supplementary material for: Dynamic full-field optical coherence tomography for live-cell imaging and growth-phase monitoring in Aspergillus fumigatus
Source: Front Cell Infect Microbiol. 2023 Jul 12;13:1183340. doi: 10.3389/fcimb.2023.1183340 (PMC10369068; doi:10.3389/fcimb.2023.1183340)
Supplement: Supplementary file 1 [file DataSheet_1.docx]

Supplementary Material

Dynamic full-field optical coherence tomography for live-cell imaging and growth phase monitoring in *Aspergillus fumigatus*

Thomas Maldiney*, Dea Garcia-Hermoso, Emilie Sitterlé, Jean-Marie Chassot, Olivier Thouvenin, Claude Boccara, Mathieu Blot, Lionel Piroth, Jean-Pierre Quenot, Pierre-Emmanuel Charles, Vishukumar Aimanianda, Bianca Podac, Léa Boulnois, Frédéric Dalle, Marc Sautour, Marie-Elisabeth Bougnoux, Fanny Lanternier

*** Correspondence:** Corresponding Author: [thomas.maldiney@ch-chalon71.fr](mailto:thomas.maldiney@ch-chalon71.fr)

# Supplementary Data

**Supplementary Video 1 (Conidial Heads.avi).** Live whole D-OCT imaging of *Aspergillus fumigatus* conidial heads on Sabouraud Dextrose Agar for 360 minutes following a 24h incubation at 30°C in a humidified chamber. This 2D-video corresponds to a 50 µm-deep axial Z-projection of the same region of interest as in Figure 1 and 2 monitored every 20 minutes for 360 minutes.

**Supplementary Video 2 (Conidiophores.avi).** Live whole D-OCT imaging of *Aspergillus fumigatus* conidiophores on Sabouraud Dextrose Agar for 180 minutes following a 24h incubation at 30°C in a humidified chamber. This 2D-video corresponds to a 50 µm-deep axial Z-projection of the same region of interest as in Figure 4 monitored every 10 minutes for 180 minutes.

**Supplementary Video 3 (Conidial head development.avi).** Live whole D-OCT imaging of *Aspergillus fumigatus* conidial head formation and development on Sabouraud Dextrose Agar for 180 minutes following a 24h incubation at 30°C in a humidified chamber. This 2D-video corresponds to a 50 µm-deep axial Z-projection monitored every 10 minutes for 180 minutes.

# Supplementary Figures

|  | **Dynamic full-field optical coherence tomography (D-FF-OCT)** | **Confocal laser scanning microscopy (CLSM)** |
| --- | --- | --- |
| **Characteristics** | conventional halogen light or LED source or with short temporal coherence length, viewable section of 1260 μm x 1260 μm, final **axial and transverse resolution of approximately 1-1.5 µm** | UV, Visible and Infrared in one system, resolution up to 8k x 8k per image, excitation range 350–1050 nm, **ultra-high optical vertical / horizontal resolution** (<< 1 µm) |
| **Pros** | practical and **easy to use for on-site clinical applications** (easy to transpose to routine clinical applications), live fungal and fungal biofilm imaging, assess the **viability of fungal cells without any preparation** nor coloration step, no need for antibody-conjugated dyes | reproducible fungal culturing format, live fungal and fungal biofilms imaging, **antibody-conjugated dyes can be used to quantitatively study live-fluorescence imaging and specific metabolic route of fungal growth**, multiple imaging wavelengths, adapted for **microscopic structures** |
| **Cons** | no conjugated dye and no specific imaging, lower resolution than CLSM, only **one imaging wavelength** | difficult to transpose to routine clinical application, less adapted for mesoscopic structures |
| **References** | - Wang L et al. Methods and applications of full-field optical coherence tomography: a review. J Biomed Opt. 2022 May;27(5):050901 - https://aquyre.com/celtivity-tm/our-technology/ | - Reichhardt C and Parsek MR. Confocal Laser Scanning Microscopy for Analysis of Pseudomonas aeruginosa Biofilm Architecture and Matrix Localization. Front Microbiol. 2019 Apr 2;10:677 - https://www.leica-microsystems.com/products/confocal-microscopes/p/leica-tcs-sp5/ |

**Supplementary Table**. Compared characteristics of dynamic full-field optical coherence tomography (D-FF-OCT) and CLSM (Confocal Laser Scanning Microscopy) technologies.


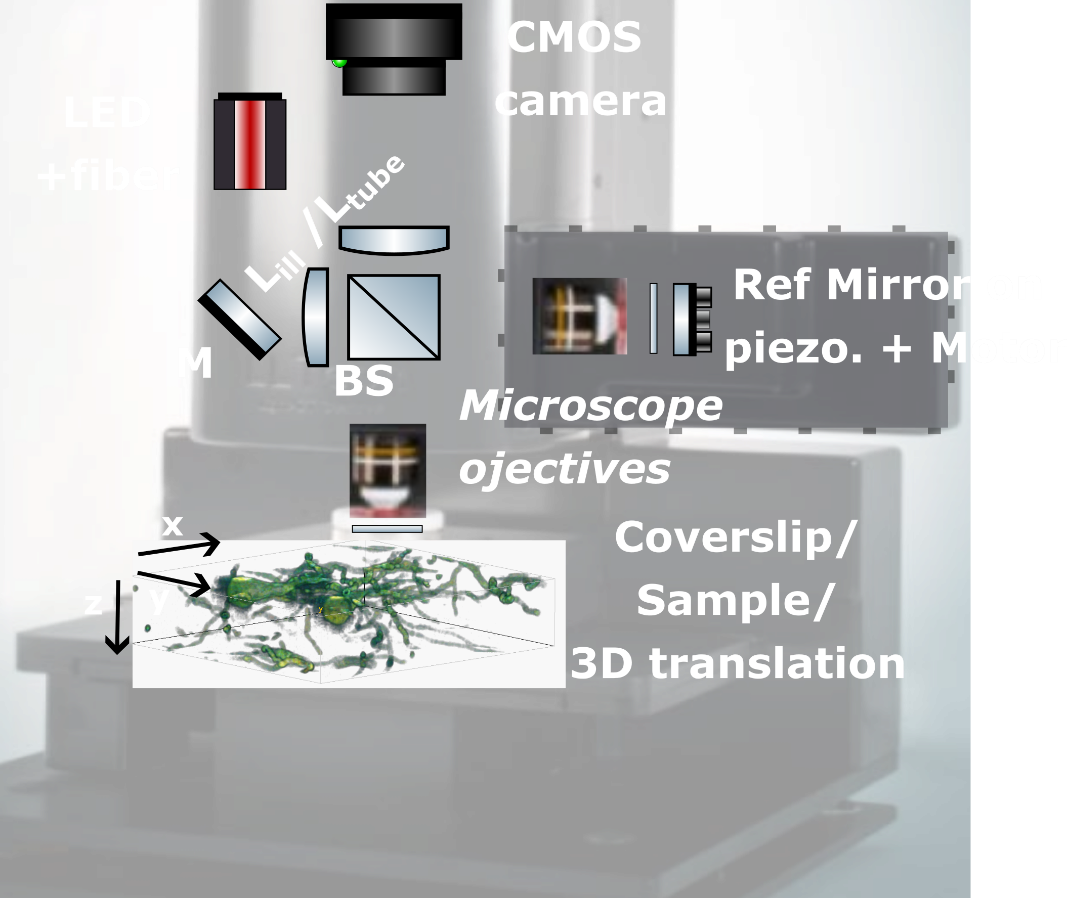


**Supplementary Figure S1**. Optical design of the Light-CT Scanner apparatus. Briefly, the system is composed of a spatially and temporally incoherent light source that illuminates a Linnik interferometer via a turning mirror (M) and a lens (L). The interferometer is composed of a beamsplitter (BS) that splits the illumination light between a sample and a reference arm. The sample arm contains a 10X, 0.3 NA microscope objective, the sample gently pressed against a 1 mm thick coverslip, as well as a 3D translation stage. The reference arm contains the same objective and the same glass coverslip but images a simple mirror instead of the sample. The reference mirror is mounted on a piezoelectric transducer to rapidly modulate the optical path difference and achieve 4 phases imaging to recover the amplitude of the interference term. The entire reference arm is mounted onto a motorized translation to adjust the coherence volume (i.e., the imaged plane in the sample). Light beams backscattered from both arms are recombined by the BS, and the image of the sample is made onto a CMOS camera thanks to a tube lens. The system design is superimposed on a picture of the actual system.


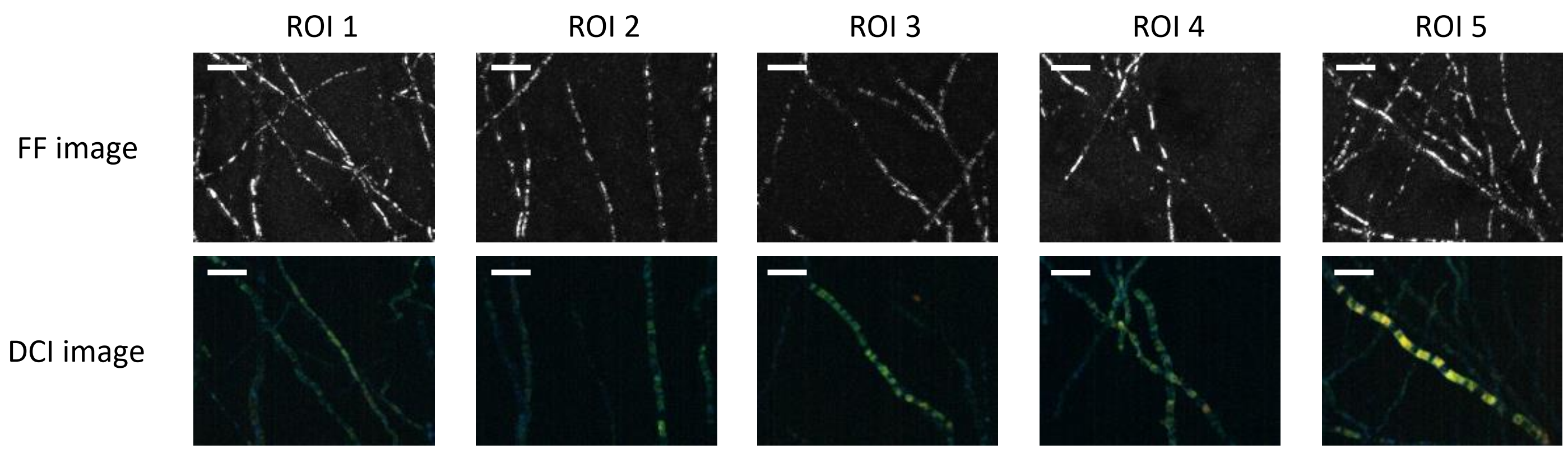


**Supplementary Figure S2.** Whole volume FF- and D-OCT imaging of *Aspergillus fumigatus* conidiophores within Sabouraud Dextrose Agar following a 24h incubation at 30°C in a humidified chamber. Images display a 50 µm-deep axial Z-projection of growing conidiophores at different stages of the developmental process within the Sabouraud Dextrose Agar. Each region of interest (ROI) shows a different metabolic stage in the developmental process of *Aspergillus fumigatus* conidiophores. Scale bar represents 25 µm. The composite RGB DCI-image translates as color each each pixel movement (red for high frequencies / fast movements, green for medium frequencies / intermediate movements and blue for low frequencies / slow movements).


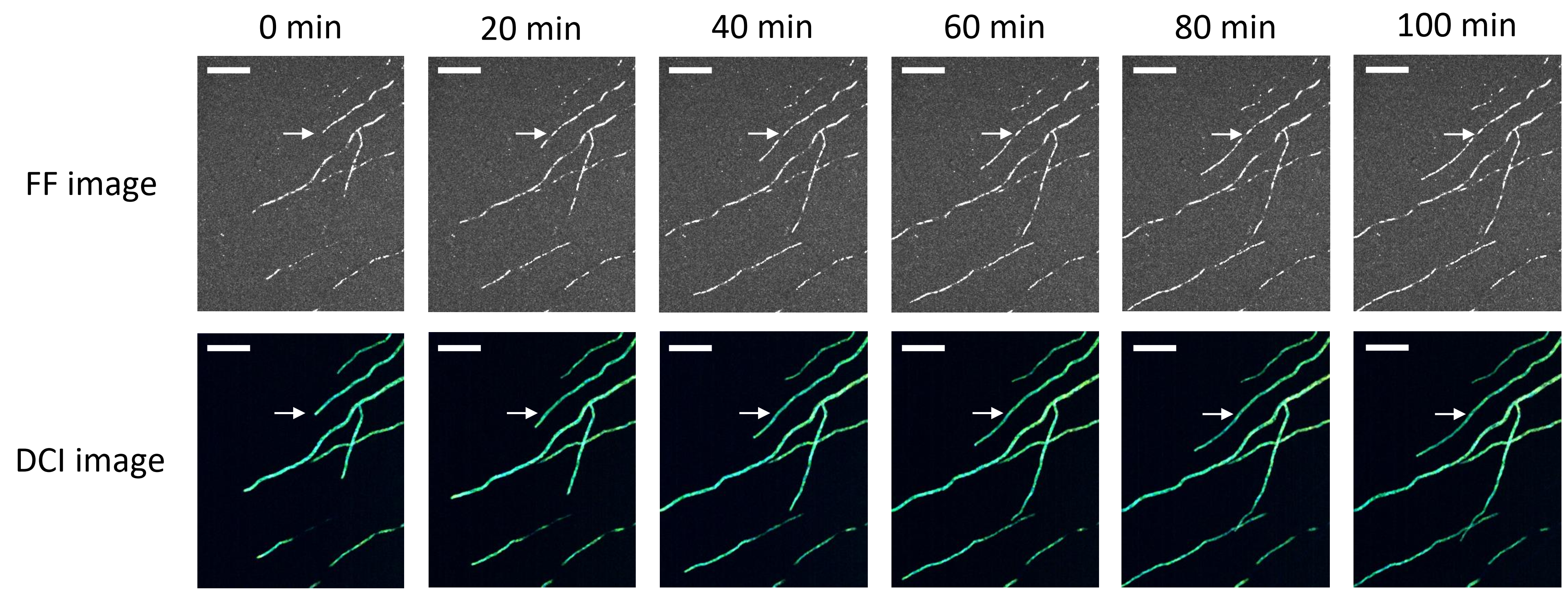


**Supplementary Figure S3.** Whole volume FF- and D-OCT live-imaging of *Aspergillus fumigatus* conidiophores growth within Sabouraud Dextrose Agar for 100 minutes following a 24h incubation at 30°C in a humidified chamber. Images display a 50 µm-deep axial Z-projection of growing conidiophores within the same volume of Sabouraud Dextrose Agar at different times (0, 20, 40, 60, 80 and 100 minutes). Scale bar represents 50 µm. The composite RGB DCI-image translates as color each each pixel movement (red for high frequencies / fast movements, green for medium frequencies / intermediate movements and blue for low frequencies / slow movements). White arrows point the growing conidiophore.


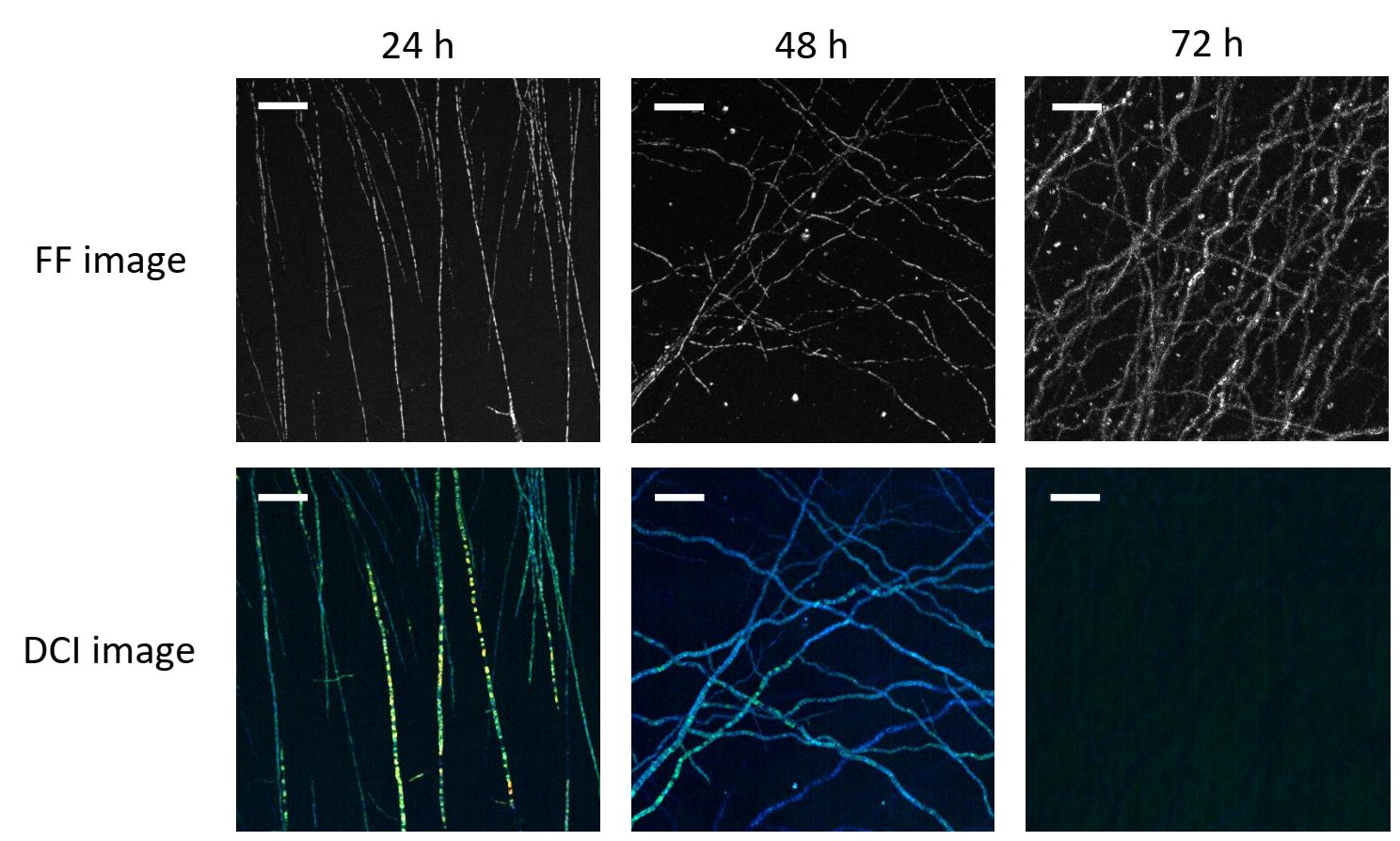


**Supplementary Figure S4.** Whole volume FF- and D-OCT imaging of *Aspergillus fumigatus* conidiophores within Sabouraud Dextrose Agar following different incubation periods (24, 48 and 72 hours) at 30°C in a humidified chamber. Images display a 50 µm-deep axial Z-projection of conidiophores within the Sabouraud Dextrose Agar. Scale bar represents 25 µm. The composite RGB DCI-image translates as color each each pixel movement (red for high frequencies / fast movements, green for medium frequencies / intermediate movements and blue for low frequencies / slow movements).


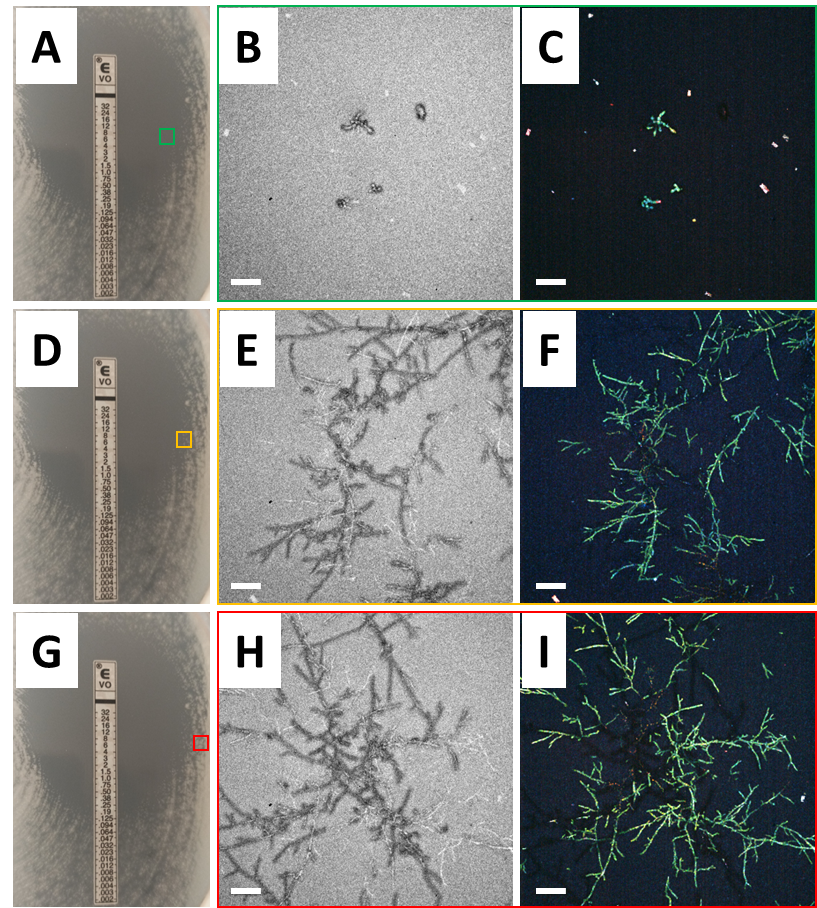


**Supplementary Figure S5.** Whole volume FF- and D-OCT imaging of Aspergillus fumigatus conidiophores within RPMI agar depending on the distance to the Etest voriconazole strip. Green square from the macroscopic image (A) shows a region of interest with high voriconazole concentration, along with the corresponding FF- (B) and D-OCT (C) images ; Orange square from the macroscopic image (D) shows a region of interest with intermediate voriconazole concentration, along with the corresponding FF- (E) and D-OCT (F) images ; Red square from the macroscopic image (G) shows a region of interest with low voriconazole concentration, along with the corresponding FF- (H) and D-OCT (I) images. D-FF-OCT images display a 50 µm-deep axial Z-projection of conidiophores within the RPMI agar. Scale bar represents 50 µm. The composite RGB DCI-image translates as color each each pixel movement (red for high frequencies / fast movements, green for medium frequencies / intermediate movements and blue for low frequencies / slow movements).


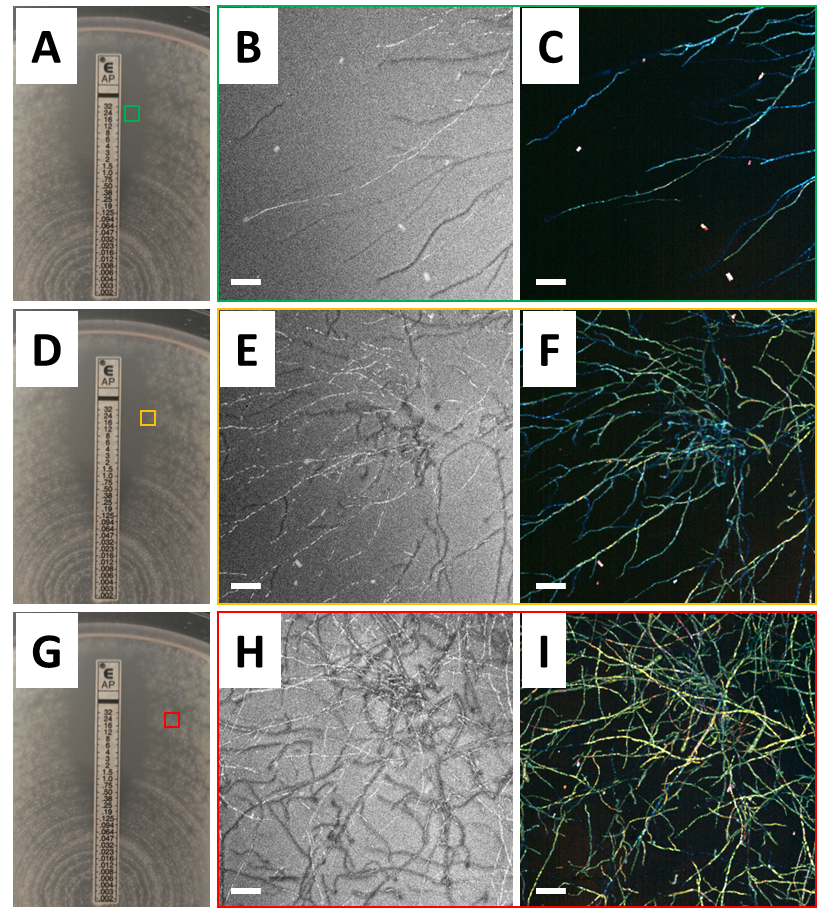


**Supplementary Figure S6.** Whole volume FF- and D-OCT imaging of Aspergillus fumigatus conidiophores within RPMI agar depending on the distance to the Etest amphotericin B strip. Green square from the macroscopic image (A) shows a region of interest with high amphotericin B concentration, along with the corresponding FF- (B) and D-OCT (C) images ; Orange square from the macroscopic image (D) shows a region of interest with intermediate amphotericin B concentration, along with the corresponding FF- (E) and D-OCT (F) images ; Red square from the macroscopic image (G) shows a region of interest with low amphotericin B concentration, along with the corresponding FF- (H) and D-OCT (I) images. D-FF-OCT images display a 50 µm-deep axial Z-projection of conidiophores within the RPMI agar. Scale bar represents 50 µm. The composite RGB DCI-image translates as color each each pixel movement (red for high frequencies / fast movements, green for medium frequencies / intermediate movements and blue for low frequencies / slow movements).


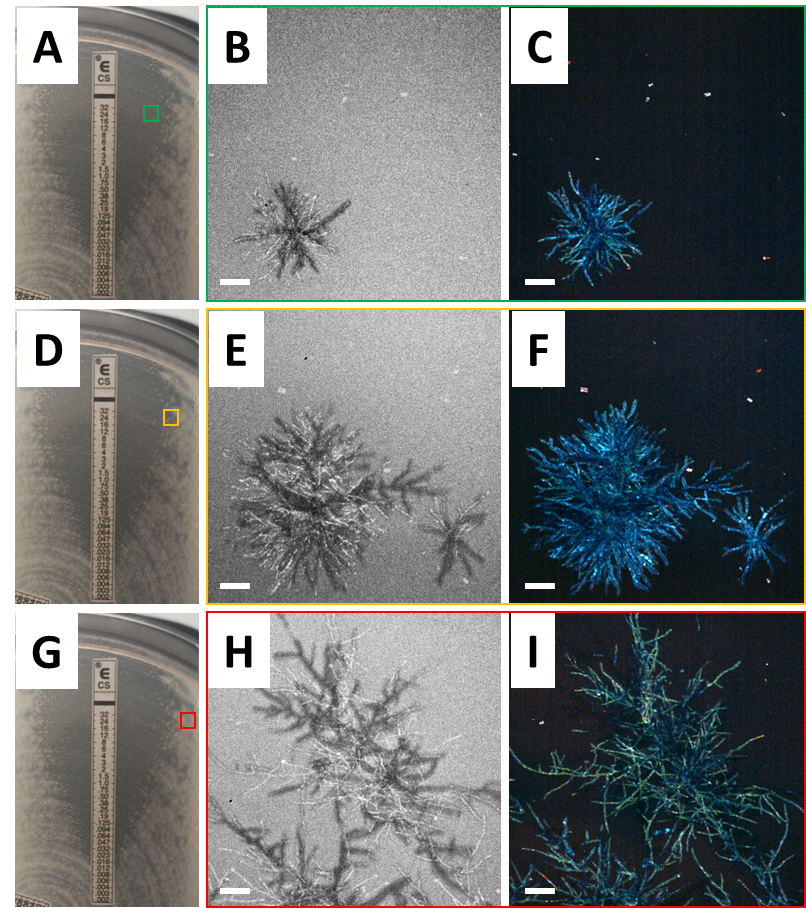


**Supplementary Figure S7.** Whole volume FF- and D-OCT imaging of Aspergillus fumigatus conidiophores within RPMI agar depending on the distance to the Etest caspofungin strip. Green square from the macroscopic image (A) shows a region of interest with high caspofungin concentration, along with the corresponding FF- (B) and D-OCT (C) images ; Orange square from the macroscopic image (D) shows a region of interest with intermediate caspofungin concentration, along with the corresponding FF- (E) and D-OCT (F) images ; Red square from the macroscopic image (G) shows a region of interest with low caspofungin concentration, along with the corresponding FF- (H) and D-OCT (I) images. D-FF-OCT images display a 50 µm-deep axial Z-projection of conidiophores within the RPMI agar. Scale bar represents 50 µm. The composite RGB DCI-image translates as color each each pixel movement (red for high frequencies / fast movements, green for medium frequencies / intermediate movements and blue for low frequencies / slow movements).
